# Comparative analysis and influential factors of embodied carbon emissions across low-rise, multi-story, and high-rise residential buildings in China

**DOI:** 10.1038/s41598-026-54989-w

**Published:** 2026-05-25

**Authors:** Jiayue Sun, Xiaocun Zhang, Fenglai Wang

**Affiliations:** 1 School of Civil Engineering, Harbin Institute of Technology, Harbin, 150090 China; 2 School of Civil & Environmental Engineering and Geography Science, Ningbo University, Ningbo, 315211 China; 3 Key Lab of Structures Dynamic Behavior and Control of the Ministry of Education, Harbin Institute of Technology, Harbin, 150090 China; 4 Key Lab Smart Prevention and Mitigation of Civil Engineering Disasters of the Ministry of Industry and Information Technology, Harbin Institute of Technology, Harbin, 150090 China

**Keywords:** Residential building, Embodied carbon intensity, Statistical analysis, Influencing factor, Comparative analysis, Engineering, Environmental sciences, Environmental social sciences

## Abstract

Embodied carbon emission reduction is crucial for promoting energy conservation and carbon reduction in residential buildings as operational carbon emissions decline. While previous studies have examined the influence of various building characteristics on embodied carbon emissions, the underlying factors responsible for the observed variations in emissions under different characteristics remain insufficiently explored. Using a dataset of 538 residential buildings in China, this study statistically analyzed the embodied carbon intensities of low-rise, multi-story, and high-rise buildings, with average values of 399.2, 442.9, and 450.1 kgCO_2e_/m^2^, respectively. Correlation analysis and significance tests were then used to examine differences in embodied carbon emissions across different characteristic categories. The results revealed that structural form and seismic fortification intensity primarily affected the embodied carbon intensity of structural materials, whereas delivery type was mainly associated with that of decorative materials. The proposed influence coefficients further indicated that concrete was the dominant factor to differences in embodied carbon intensity associated with structural forms and seismic fortification intensity in low-rise and high-rise buildings, whereas steel made a greater contribution in multi-story buildings. Moreover, the analysis of potential carbon reduction measures indicated that low-rise and multi-story buildings with frame structures exhibited the highest reduction potential, with reductions of 13.9% and 15.2%, respectively, achievable through material consumption optimization and transport distance reduction. However, in high-rise buildings, shear wall structures exhibited the highest reduction potential (12.5%). This study provides practical insights into optimizing residential building design from a low-carbon perspective.

## Introduction

The building and construction industry is an key area for energy conservation and emission reduction^1^, accounting for 37% of the total energy and process-related carbon dioxide emissions^2^. As the world’s largest carbon emitter^3^, China announced its strategic goals of achieving carbon peak and carbon neutrality in 2020 to contribute to global climate change mitigation. Subsequently, a series of policies promoting green, low-carbon, and sustainable development have been issued across various industries to support the achievement of these targets. According to relevant statistics, building construction and operation are responsible for 50% of total energy consumption and approximately 33% of total carbon emissions in China^4^. Therefore, it is crucial to save energy and reduce carbon emissions in the building and construction industry. Meanwhile, data from the China Statistical Yearbook on Construction^5^ revealed that residential buildings consistently accounting for the largest share of annual newly constructed areas among all building types, and their associated carbon emissions typically exceed those of public buildings^6^. In this context, carbon emissions from residential buildings deserve particular attention within the built environment.

Over the building life cycle, which includes production, construction, operation, and disposal phases, carbon emissions mainly arise from production and operational activities. The relative contribution of embodied carbon emissions from the material production phase has increased^7–9^, as operational emissions have been progressively reduced via the application of clean energy and low-carbon technologies^10–12^. Moreover, previous studies have shown that although operational emissions account for the greatest share of total life cycle impacts^13^, the carbon emission intensity during the construction stage can substantially exceed that during the operation stage when assessed from a temporal perspective^14,15^. Hence, there is significant potential for reducing embodied carbon emissions, and the considerable construction volume in China highlights the importance of implementing low-carbon construction for residential buildings. Such efforts are essential for promoting sustainable development in the construction sector.

## Literature review

With respect to previous studies on embodied carbon emissions, most scholars have evaluated the embodied carbon emissions of individual buildings and compared the relative contributions of different subprocesses. Li et al.^16^ analyzed the carbon emissions associated with prefabricated buildings and emphasized that the production of precast component is a major source of embodied carbon emissions. Liu and Huang^17^ indicated that the material production phase accounted for nearly 88% of the embodied carbon emissions from prefabricated buildings. Consistent with these findings, other researchers have reported similar conclusions^18–20^.

Considering the large variability in embodied carbon emissions of individual buildings, recent studies have increasingly focused on analyzing multiple building cases^21,22^, thereby revealing the statistical characteristics of embodied carbon emissions. Zhang et al.^23^ suggested that the embodied carbon emissions per floor area (i.e., the carbon emission intensity) of high-rise residential buildings followed an approximately lognormal distribution function on the basis of a database encompassing 403 buildings. Luo et al.^24^ examined the carbon emissions of 78 office buildings and reported a positive correlation between the carbon emission intensity and building height. Moreover, based on a statistical analysis of 95 residential building cases, Chastas et al.^25^ reported that the embodied carbon emissions ranged from 179.3 to 1050 kgCO_2e_/m². Roh et al.^26^ analyzed the material inventories of 443 apartment buildings in South Korea and found that their embodied carbon emissions ranged between 357 and 418 kgCO_2e_/m². These findings indicate substantial cross-country variations in embodied carbon, which may arise from differences in system boundaries, calculation methods, and national development contexts.

With the contribution of embodied carbon emissions from the production phase steadily increasing throughout the entire building life cycle^27^, an increasing number of scholars have proposed that reducing embodied carbon emissions is key to advancing low-carbon building practices. Consequently, the factors influencing embodied carbon emissions have been explored in depth.

Researchers have explored the factors influencing embodied carbon emissions using comparative analysis methods. Zhang and Zheng^28^ and Cang et al.^29^ compared embodied carbon emissions across different structural forms, thereby verifying their individual impacts. Other researchers have compared buildings with different heights^30^. Machado et al.^31^ indicated that the main driving factor of embodied emissions is the structural weight. Moreover, considering the impact of different construction techniques, Zhao et al.^32^ assessed the embodied carbon emissions of traditional, prefabricated, and green buildings. Their findings highlighted carbon emission differences among various techniques. Other scholars have adopted scenario analysis approaches to identify the main factors influencing embodied carbon emissions. Hong et al.^33^ considered steel, concrete, talcum powder, cement, aluminum, and glass as the primary materials influencing embodied carbon emissions via scenario analysis. Comparatively, the transportation distance and prefabrication rate were identified as the major factors influencing the embodied carbon emissions of prefabricated buildings^34–36^. Additionally, sensitivity analysis has been employed to identify the factors influencing embodied carbon emissions^19,37,38^. The most relevant results indicate that the main contributor to carbon emissions is the consumption of steel, concrete, cement, and formwork^39–41^.

On the basis of the identified influencing factors for the building production phase, emission reduction measures involving different pathways have been studied, such as reducing emission factors and the consumption of materials. With respect to emission factors, efforts to reduce carbon emissions have focused on applying low-carbon materials or increasing the recycling rate^42–44^. Researchers have stated that increasing the material recycling rate is one of the most effective measures for reducing emissions in the short term^45,46^. To minimize material consumption in building construction, low-carbon structure design optimization has been proposed^47–49^. Other scholars have noted that the use of high-strength materials is a viable approach for reducing material inputs^50,51^. However, limited studies have provided emission reduction measures for the transportation process, such as optimizing transportation routes and adopting new energy transportation vehicles^44,52,53^.

Although embodied carbon emissions during the production phase have been extensively investigated, several knowledge gaps remain. First, studies on embodied carbon emissions at the individual-building level are often case-specific because of differences in system boundaries, data sources, calculation methods, and national development level^17–19^. While recent studies have statistically analyzed building sample datasets, they have mainly focused on the effects of characteristic parameters on carbon emissions^24,26,31^. However, the driving forces underlying differences in embodied carbon emissions across various characteristic categories require further investigated. Second, although carbon reduction measures have been proposed for residential buildings, differences in the performance of reduction measures under the influence of various building characteristic parameters have not been sufficiently considered^54–56^.

To address the above knowledge gaps, data from 538 residential buildings covering a broad range of building characteristics were collected. The corresponding embodied carbon emissions were analyzed to identify the key building characteristic parameters and the underlying drivers of emission differences. Accordingly, the primary contributions of this study are as follows: (1) a comparative analysis of embodied carbon intensities across low-rise, multi-story, and high-rise residential buildings was conducted to discern differences in their statistical characteristics; (2) the driving forces underlying differences in embodied carbon emission intensity across different building characteristic categories were analyzed using the proposed influence coefficients; and (3) carbon mitigation measures were proposed for residential buildings with different heights, providing practical references for choosing materials and optimizing design schemes.

## Methodology

### Research scope

**Fig. 1:**
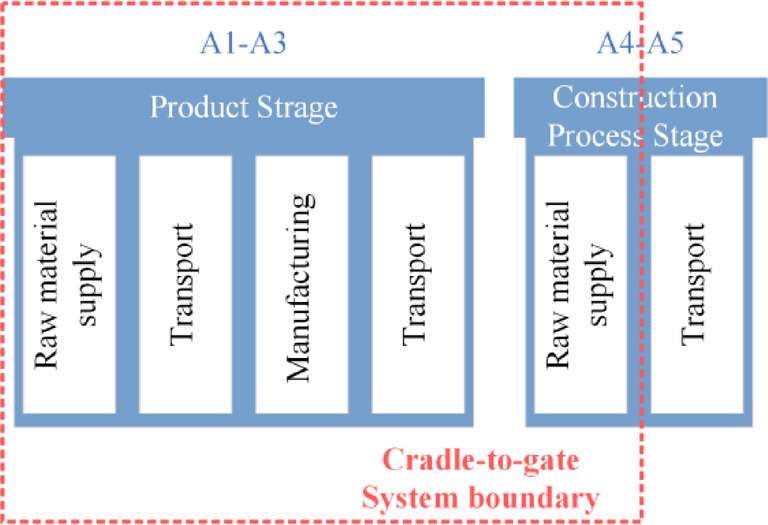
The cradle-to-site system boundary adopted in this study.

In previous studies, the material production and transportation phases have been proven to be the primary sources of building embodied carbon emissions^18,21,57^. Therefore, a cradle-to-site system boundary was adopted to assess the embodied carbon emissions of residential buildings, corresponding to stages A1–A4 defined in EN 15978:2011^58^, as illustrated in Fig. 1.

**Fig. 2:**
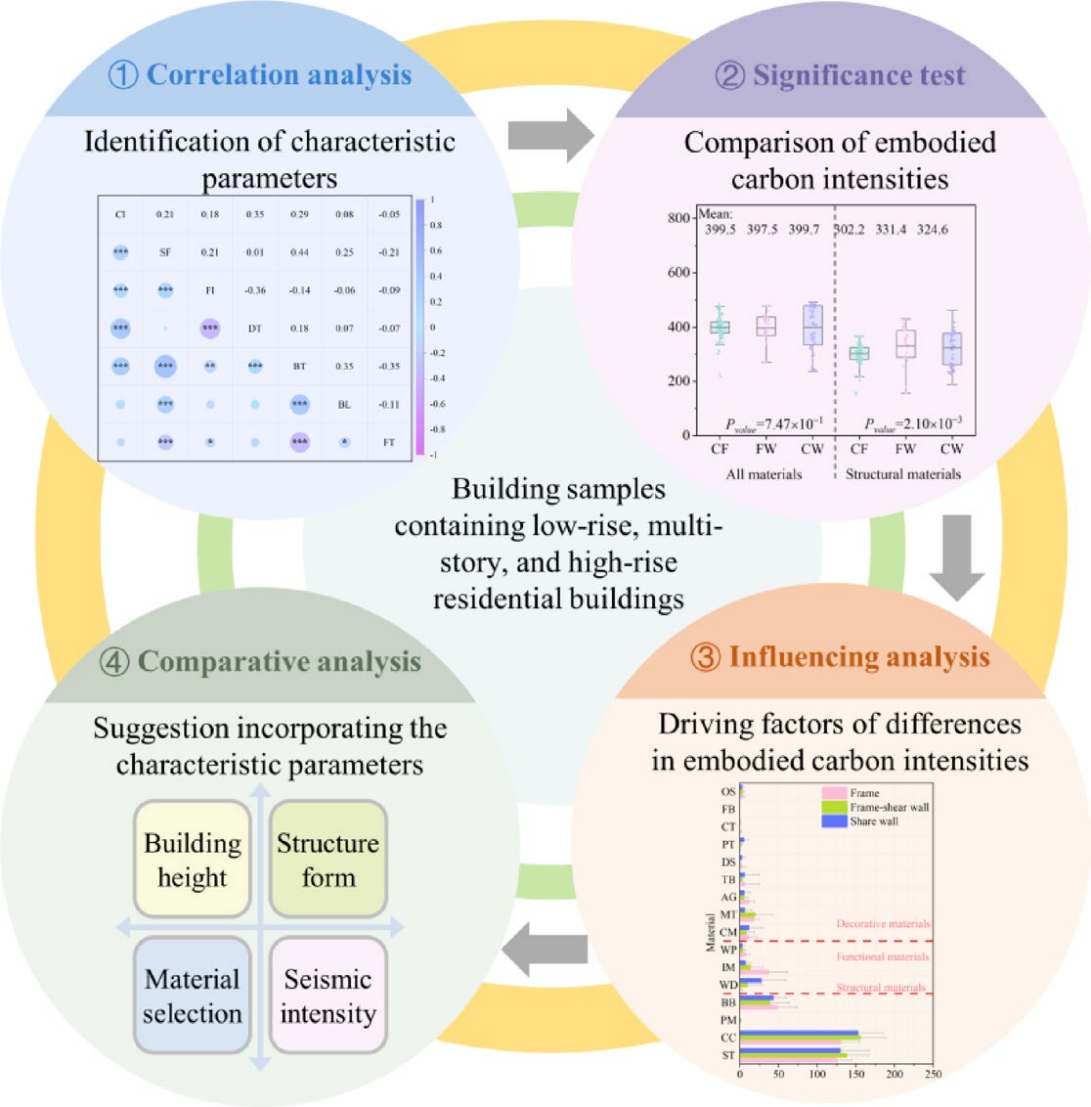
Research framework.

To facilitate comparisons among different buildings, 1 m^2^ building floor area has been determined as the functional unit in national standards^59^ and prior studies^60,61^. Therefore, to ensure consistency with most previous studies, this study employed unit floor area as the functional unit for quantifying building embodied carbon emissions. The research framework is illustrated in Fig. 2. First, according to the defined system boundary, the embodied carbon intensities of 538 residential buildings were assessed. On the basis of correlation analysis, building characteristic parameters associated with embodied carbon intensity were identified. Statistical analysis was then conducted to describe the distribution of embodied carbon intensity in low-rise, multi-story, and high-rise residential buildings. Subsequently, the influence of different characteristic parameters on building embodied carbon intensity were evaluated using significance testing. Finally, based on the coefficients of influence relevant to materials, the driving forces influencing the differences in embodied carbon intensity under different classifications of building characteristic parameters were analyzed. Bases on the above analysis, corresponding carbon reduction strategies for residential buildings with different heights were compared by considering the selection of various materials and structures.

### Embodied carbon intensity assessment

Considering building volume differences, the embodied carbon intensity, which is defined as the embodied carbon emissions per floor area, can be evaluated as follows:

1 $$C{I_{{\mathrm{M}},i}}=\frac{{{E_{{\mathrm{M}},i}}}}{{{A_i}}}$$

where $C{I_{{\mathrm{M}},i}}$, ${E_{{\mathrm{M}},i}}$ and ${A_i}$ are the embodied carbon intensity, embodied carbon emissions, and floor area, respectively, of building *i*.

Notably, the total embodied carbon emissions originating from materials can be calculated as:

2 $${E_{{\mathrm{M}},i}}={E_{{\mathrm{mat}},i}}+{E_{{\mathrm{tra}},i}}=\sum\limits_{j} {{Q_{{\mathrm{M}},ji}} \cdot ({F_{{\mathrm{M}},ji}}+{F_{{\mathrm{T}},ji}} \cdot {D_{{\mathrm{M}},ji}})}$$

where ${E_{{\mathrm{mat}},i}}$ and ${E_{{\mathrm{tra}},i}}$ are the carbon emissions from the material production and transportation processes, respectively; ${Q_{{\mathrm{M}},ji}}$ is the weight of building material *j* in building *i*; ${F_{{\mathrm{M}},ji}}$ and ${F_{{\mathrm{T}},ji}}$ are the emission factors of material *j* and transportation, respectively; and ${D_{{\mathrm{M}},ji}}$ is the transport distance of material *j* in building *i*.

The embodied carbon intensity can be calculated via Eq. (2) as follows:

3 $$C{I_{{\mathrm{M}},i}}=\sum\limits_{j} {{I_{{\mathrm{M}},ji}} \cdot ({F_{{\mathrm{M}},j}}+{F_{{\mathrm{T}},j}} \cdot {D_{{\mathrm{M}},ji}})}$$

where ${I_{{\mathrm{M}},ji}}$ is the consumption of material *j* per square meter of floor area in building *i*, which is defined as the material intensity in this study.

### Correlation analysis

Previous studies have indicated that various building characteristics—such as building height, structural form, seismic fortification intensity, delivery type, foundation type—may influence embodied carbon intensity^23,29^. To identify the key factors affecting embodied carbon intensity, this study conducted a correlation analysis to examine the relationships between individual building characteristics and embodied carbon intensity, as well as the intercorrelations among different characteristics.

Correlation analysis primarily included Pearson’s correlation coefficient and Spearman’s rank correlation coefficient^62^. Notably, Spearman correlation analysis can be applied to assess correlations among non-normally distributed data and discrete variables^63^, and relevant studies have revealed that the embodied carbon emissions of residential buildings may not adhere to a normal distribution^64,65^. Therefore, the Spearman rank correlation coefficient method was adopted instead.

### Statistical analysis

On the basis of the obtained 538 residential building samples, carbon emission intensities categorized by different building characteristic parameters, carbon emission distributions and their differences were analyzed. Descriptive and inferential statistical indicators were adopted to clarify the statistical characteristics and magnitude of the differences in embodied carbon emissions. Generally, descriptive statistical indicators such as the mean, median, and standard deviation are fundamental in explaining the concentration trend and degree of discreteness of data. They also fulfill a crucial role in identifying outliers. In comparison, inferential statistical indicators such as the standard error and confidence interval reflect the reliability of sample inference to the population. Hence, the mean, median, standard deviation, standard error, and confidence interval were adopted to evaluate the characteristics of embodied carbon intensities considering the samples clustered by different building characteristic parameters.

### Significance test

To examine the potential effects of different building characteristic parameters on embodied carbon intensity, the Kruskal‒Wallis rank-sum test was further applied to evaluate differences in embodied carbon intensity among different characteristic categories within low-rise, multi-story, and high-rise buildings. The Kruskal‒Wallis rank-sum test is a nonparametric test applicable to multiple independent samples without assumptions regarding the sample distribution. Unlike the t-test and analysis of variance method, which generally require normality and homogeneity of variance, the Kruskal‒Wallis test is suitable for non-normally distributed data. Because the selected building characteristic parameters were categorical grouping variables and the corresponding embodied carbon intensity distributions did not necessarily satisfy normality, the Kruskal‒Wallis rank-sum test was applied to analyze the embodied carbon emission differences caused by the building characteristic parameters at a significance level of 5%.

### Influencing factor analysis

On the basis of the significance test results, the coefficient of influence was proposed to better understand the driving forces underlying the differences in embodied carbon emissions due to various building characteristic parameters. The coefficient of influence was defined as the contribution of a certain type of material to the total difference in embodied carbon emissions, which can be obtained as:

4 $${r_j}=\frac{{\left| {\Delta {E_j}} \right|}}{{\sum\limits_{j} {\left| {\Delta {E_j}} \right|} }}$$

5 $$\Delta {E_j}=\frac{1}{m}\sum\limits_{m} {{E_{j,m}}} - \frac{1}{n}\sum\limits_{n} {{E_{j,n}}}$$

where $\Delta {E_j}$ is the difference in the embodied carbon intensity attributed to material *j*; ${E_{j,m}}$ and ${E_{j,n}}$ are the carbon emission intensities of material *j* in clusters *m* and *n*, respectively; and *m* and *n* are the numbers of sample buildings in the different clusters. Moreover, the differences in carbon emissions can directly reveal the within-cluster carbon emission differences of material *j* incorporating various building characteristic parameters.

## Data collection

### Data sources and processing

Statistical analysis was adopted to explore the general characteristics of the embodied carbon intensity in residential buildings. To ensure diversity of the building samples, residential buildings with different building characteristics, such as structural form, building type, delivery type, and seismic fortification intensity, were considered. Various residential building samples were subsequently obtained from research reports and field investigations. Material consumption data and basic information for building samples provided by field investigations were acquired from engineering documents, including building design schemes and bills of quantities, whereas data for the other samples were directly obtained from research reports relevant to Chinese building cases.

Notably, the collected building samples were obtained from different sources, which may lead to missing data and outliers during the acquisition and recording processes. To ensure the quality of the dataset and minimize the impact of outliers on the analytical results, the raw data were processed according to the following data cleaning criteria:

- The quartile method^66,67^ was adopted to identify outliers in the collected samples, which were subsequently excluded from the dataset.
- Samples lacking essential information on building characteristics and indicating an obvious bias in material consumption were eliminated.
- The average imputation method^68^ was employed to address samples with partially missing material consumption data.

### Basic information

Based on the above criteria, a final dataset comprising 538 residential building samples was established. According to the height classification criteria specified in the current Chinese standard^69^, the dataset comprised 180 low-rise buildings, 177 multi-story buildings, and 181 high-rise buildings. Among these building categories, low-rise buildings included townhouses, semi-detached villas, and buildings with no more than three stories. Multi-story buildings were defined as those with a height below 27 m and fewer than nine stories, whereas buildings higher than 27 m were classified as high-rise buildings.

**Fig. 3:**
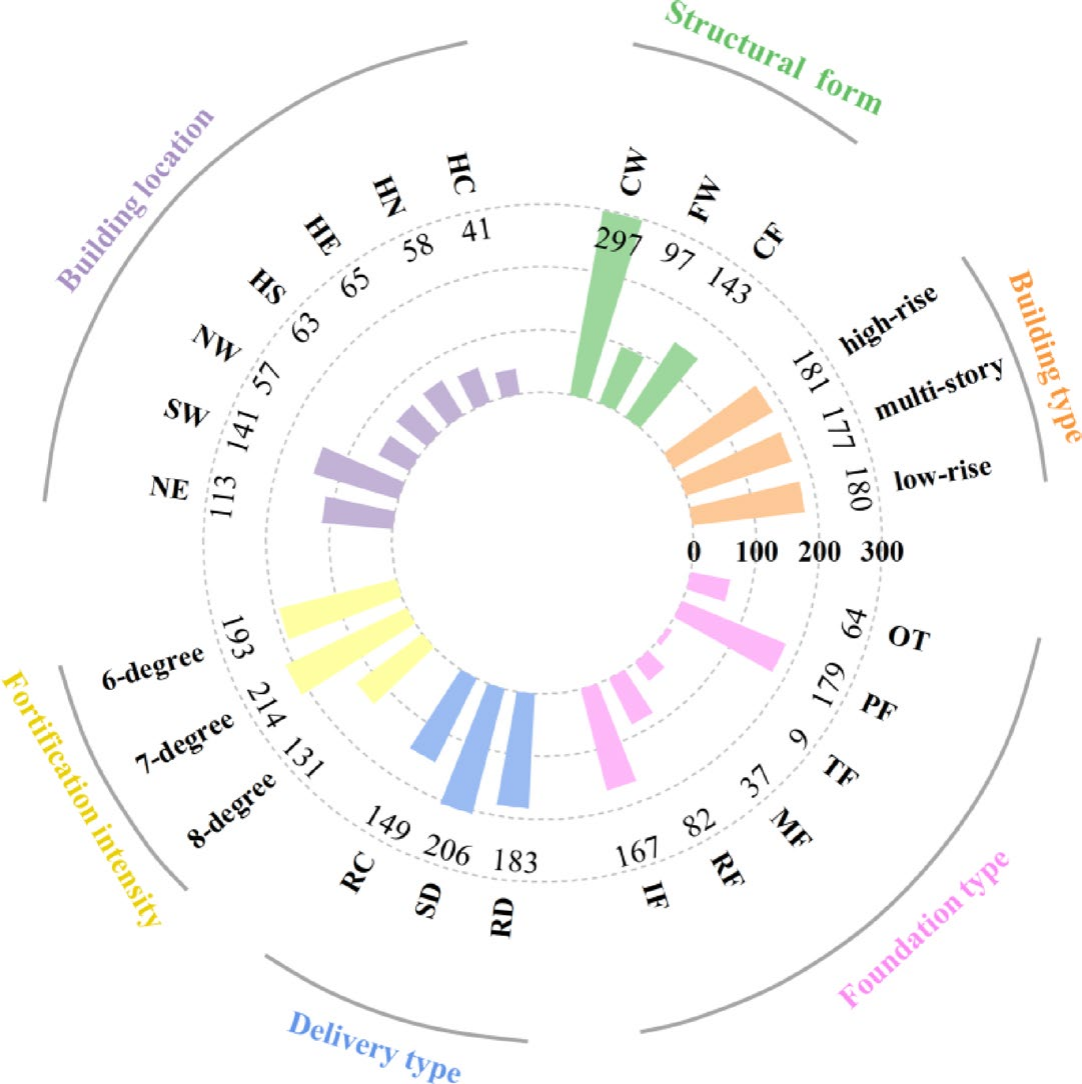
Overview of the 538 residential buildings.

Moreover, information on building characteristics and material inventories was collected for each sample. The distribution of the building samples is shown in Fig. 3, covering major building characteristics, including structural form, building type, foundation type, delivery type, seismic fortification intensity, and building location. Among these characteristics, building location indicates the regional distribution of the collected samples. As shown in Fig. 3, the samples covered seven geographical regions in China, with the number of samples in each category indicated in the figure. These characteristics reflect differences in structural systems, foundation conditions, delivery requirements, seismic design levels, and regional contexts. Therefore, the sample size of 538 buildings and the coverage of multiple building characteristics provide a practically relevant empirical basis for the statistical analyses conducted in this study.

### Material intensity

To assess the embodied carbon intensity of the residential building samples, the consumption of commonly used building materials in each sample was determined from the bill of quantities. Material intensity, defined as material consumption divided by building floor area Eq. (3), was assessed to establish the inventory for embodied carbon assessment. Notably, both material consumption and material type may vary considerably across different building design schemes. Considering data consistency and availability, 16 types of materials were selected based on the following factors:

(1) Materials that are commonly used or consumed in large quantities in buildings; (2) materials that make substantial contribute to carbon emissions^1,70,71^; (3) materials specified in national standards^59^. Among the selected building materials, steel, concrete, prefabricated structural members, and bricks and blocks were included in structural materials; cement, mortar, aggregate, timber, decorative plates, painting, ceramic tiles, floorboards and other decorative materials were categorized as decorative materials; and windows and doors, insulation materials, and waterproof materials were defined as functional materials. Given that the material intensity of a given building sample may be influenced by design details in different geographical regions, Table 1 presents the corresponding descriptive statistics to delineate the scope of the analysis.

**Table 1:** Statistics of building material intensities for the 538 residential buildings (kg/m^2^).

No.	Material	Range	Mean	Median	Standard deviation
1	Steel (ST)	17.6–115.8	57.9	56.3	15.1
2	Concrete (CC)	382.6–1927.0	1039.6	1034.9	233.6
3	Prefabricated member (PM)	0–516.9	19.7	0	64.6
4	Brick and block (BB)	0–758.5	93.7	80.5	59.5
5	Cement (CM)	0–170.5	21.8	9.8	24.3
6	Mortar (MT)	0–484.6	69.8	58.5	70.5
7	Aggregate (AG)	0–1099.8	108.2	69.3	151.0
8	Timber (TB)	0–178.8	7.0	4.6	14.5
9	Window and door (WD)	0–16.4	3.5	1.7	3.8
10	Insulation material (IM)	0–81.5	11.3	2.0	21.2
11	Waterproof material (WF)	0–22.6	3.0	2.4	2.9
12	Decorative plate (OP)	0–11.7	0.9	0.2	1.7
13	Painting (PT)	0–13.5	1.4	0.4	2.6
14	Ceramic tile (CT)	0–61.1	7.5	0.6	12.9
15	Floorboard (FB)	0–4.8	0.2	0.0	0.3
16	Others (OS)	0–32.9	1.1	0.2	2.7

### Emission factors

The carbon emission factors of materials are essential basic data for assessing the embodied carbon intensity of buildings, and the reliability and consistency of the associated data sources directly influence the accuracy and comparability of the assessment results. Hence, official data from the Chinese standard for building carbon emission calculations^59^ were utilized as the primary reference data. However, for material types not included in the standard, emission factors reported in previous studies^28,39^ were used to supplement the dataset. Moreover, the emission factor of transportation was assumed as 0.179 kgCO_2e_/(t·km), as specified in the relevant Chinese standard^59^ for medium-sized diesel trucks. Table 2 provides a summary of the reference values for the emission factors of the materials considered.

**Table 2:** Reference values for emission factors of building materials.

No.	Specification of materials	Unit	Reference value (kgCO_2e_/unit)	Source
1	Steel bar	t	2340	MHURD (2019)
	Section steel	t	2310	MHURD (2019)
2	Concrete C20	m^3^	265	Zhang et al. (2020)
	Concrete C25	m^3^	293	Zhang et al. (2023)
	Concrete C30	m^3^	316	Zhang et al. (2023)
	Concrete C35	m^3^	363	Zhang et al. (2023)
3	Prefabricated member	m^3^	720	Zhang et al. (2023)
4	Shale solid brick	m^3^	292	MHURD (2019)
	Shale hollow brick	m^3^	204	MHURD (2019)
	Concrete solid brick	m^3^	235	Zhang et al. (2020)
	Concrete hollow brick	m^3^	336	Zhang et al. (2020)
	Clay brick	m^3^	295	MHURD (2019)
	Concrete hollow block	m^3^	180	Zhang et al. (2020)
	Aerated concrete block	m^3^	270	Zhang et al. (2020)
	Gangue porous brick	m^3^	18	MHURD (2019)
5	Cement	t	735	MHURD (2019)
6	Mortar	m^3^	234	Zhang et al. (2023)
7	Gravel	t	2.18	MHURD (2019)
	Sand	t	2.51	MHURD (2019)
8	Timber	m^3^	178	Zhang et al. (2023)
9	Window and door	m^2^	194	MHURD (2019)
10	EPS insulation material	kg	5.02	MHURD (2019)
	XPS insulation material	kg	6.12	MHURD (2019)
11	Rolled waterproof	m^2^	0.54	Zhang et al. (2023)
12	Calcium silicate plate	m^2^	1.8	Zhang et al. (2023)
	Aluminum buckle plate	m^2^	32.9	Zhang et al. (2023)
	Gypsum plate	m^2^	4.4	Zhang et al. (2023)
13	Painting	kg	3.5	Zhang et al. (2024)
14	Ceramic tile	m^2^	13.3	Zhang et al. (2023)
15	Floor board	m^2^	2.9	Zhang et al. (2023)
16	Keel	kg	4.3	Zhang et al. (2023)
	Railing	kg	1.8	Zhang et al. (2024)
	Handrail	m	9.6	Zhang et al. (2023)
	Decorative stone	m^2^	65.6	Zhang et al. (2023)
	partition	m^2^	90.2	Zhang et al. (2023)

## Results and discussion

### Overview of the embodied carbon intensity levels

#### Main building characteristic parameters

**Fig. 4:**
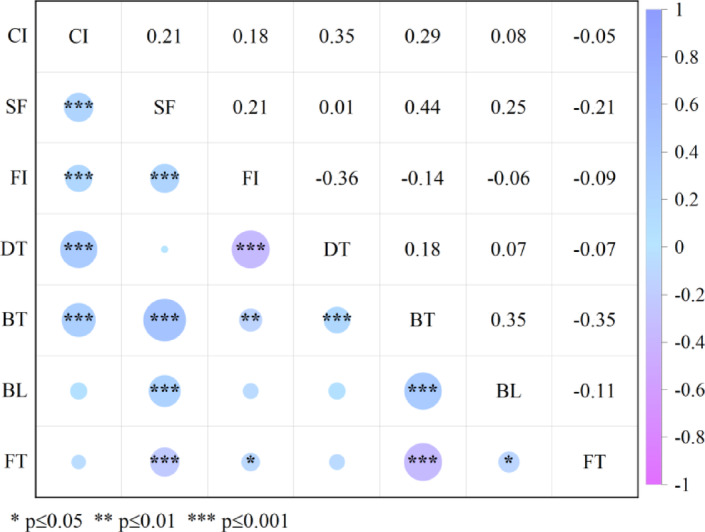
Spearman rank correlation analysis of characteristic parameters. “CI”, “SF”, “FI”, “DT”, “BT”, “BL”, and “FT” represent the embodied carbon intensity, structural form, seismic fortification intensity, delivery type, building type, building location, and foundation type, respectively.

The correlations between the embodied carbon intensity and building characteristic parameters were analyzed using the Spearman correlation coefficient, and the results are shown in Fig. 4. At the 5% significance level, the building location and foundation type were observed to have no statistically significant relationship with embodied carbon intensity. Therefore, building type, structural form, seismic fortification intensity, and delivery type were considered the primary building characteristic parameters for subsequent analysis.

It should be noted that building location was not a dominant direct factor affecting embodied carbon intensity in the present dataset, rather than implying that regional conditions are irrelevant to embodied carbon emissions. Regional differences may indirectly influence embodied carbon emissions by affecting climate-related envelope material requirements, insulation material use, window and door system selection and seismic fortification requirements.

#### Statistics on the embodied carbon intensity

Previous studies on building height and embodied carbon intensity have revealed that the number of floors influences the embodied carbon intensity of residential buildings^72,73^. Hence, the 538 residential building cases were first classified into low-rise, multi-story and high-rise buildings according to the Chinese Uniform Standard for Design of Civil Buildings^69^. The corresponding descriptive statistics of embodied carbon intensity are summarized in Table 3.

**Table 3:** Statistics of embodied carbon intensities for 538 residential buildings across different building types.

Carbon intensity (kg CO_2e_/m^2^)	Statistics	Low-rise building	Multi-story building	High-rise building
Material production (A1-A3)	Range	[180.9, 461.1]	[276.4, 596.3]	[320.8, 621.5]
	Mean	**359.7**	**391.2**	**409.6**
	Median	365.3	389.1	409.6
	Standard deviation	59.2	65.9	56.0
	Confidence interval (95%)	[351.1, 368.4]	[381.5, 400.9]	[401.4, 417.7]
Material transportation (A4)	Range	[13.5, 80.3]	[15.4, 141.0]	[18.5, 114.6]
	Mean	**39.5**	**51.7**	**40.6**
	Median	39.4	44.9	39.5
	Standard deviation	11.6	23.1	11.8
	Confidence interval (95%)	[37.8, 41.2]	[48.2, 55.1]	[38.8, 42.3]
Materialization phase (A1-A4)	Range	[217.6, 492.8]	[292.4, 652.9]	[350.2, 664.4]
	Mean	**399.2**	**442.9**	**450.1**
	Median	409.1	438.6	442.2
	Standard deviation	59.7	75.9	59.5
	Confidence interval (95%)	[390.5, 407.9]	[431.7, 454.1]	[441.5, 458.8]

Across the three building height categories, the mean embodied carbon intensities were 399.2, 442.9, and 450.1 kgCO_2e_/m^2^ for low-rise, multi-story, and high-rise buildings, respectively, indicating an overall increasing trend with building height. During the material production stage, the average embodied carbon intensity of high-rise buildings was comparable to that of multi-story buildings, but approximately 13.9% higher than that of low-rise buildings. During the transportation stage, the average embodied carbon intensities of low-rise, multi-story, and high-rise buildings were 39.5, 51.7, and 40.6 kgCO_2e_/m^2^, respectively. Compared with low-rise and high-rise buildings, multi-story buildings exhibit slightly higher total material consumption. In particular, their consumption of heavier materials, including aggregates and prefabricated members, was greater than that of high-rise buildings, contributing to slightly higher transportation carbon emissions.

Overall, for low-rise, multi-story, and high-rise buildings, the material production stage accounted for average contributions of 90.1%, 88.3% and 91.0% of embodied carbon emission intensity, respectively, highlighting its dominant contribution. Notably, the coefficients of variation for embodied carbon intensity during the transportation stage were higher than those during the production stage, reaching 0.29, 0.45, and 0.29 for low-rise, multi-story, and high-rise buildings, respectively. Therefore, the transportation stage contributed substantially to the variability in the embodied carbon intensity.

**Fig. 5:**
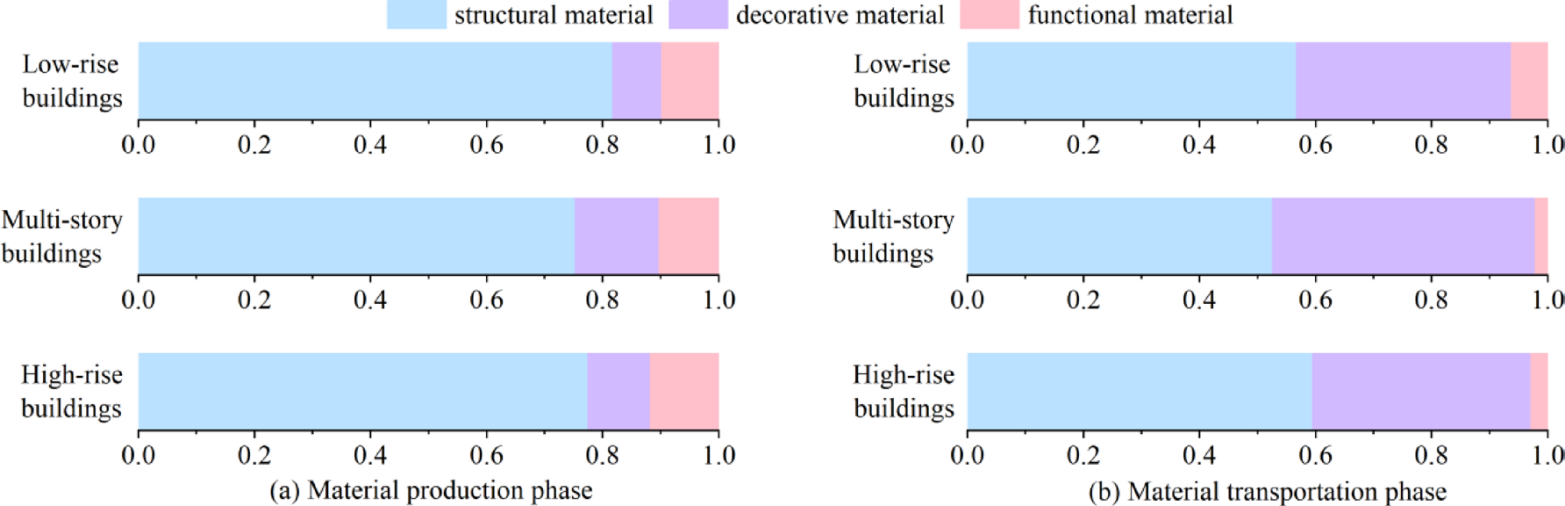
Contribution of structural, decorative and functional materials to embodied carbon intensities in low-rise, multi-story, and high-rise buildings.

Moreover, Fig. 5 compares the relative contributions of structural, decorative, and functional materials to embodied carbon intensity among low-rise, multi-story, and high-rise buildings. As shown in Fig. 5a, structural materials accounted for the largest share of embodied carbon intensity during the material production phase, with average contributions of 81.7%, 75.3%, and 77.5% in low-rise, multi-story, and high-rise buildings, respectively. In contrast, decorative and functional materials made relatively similar contributions during this phase. Figure 5b illustrates that structural and decorative materials were the primary contributors to embodied carbon intensity during the transportation phase. Notably, the contribution of decorative materials increased markedly compared with that during the production phase, reaching 37.0%, 45.3%, and 37.6% in low-rise, multi-story, and high-rise buildings, respectively.

**Fig. 6:**
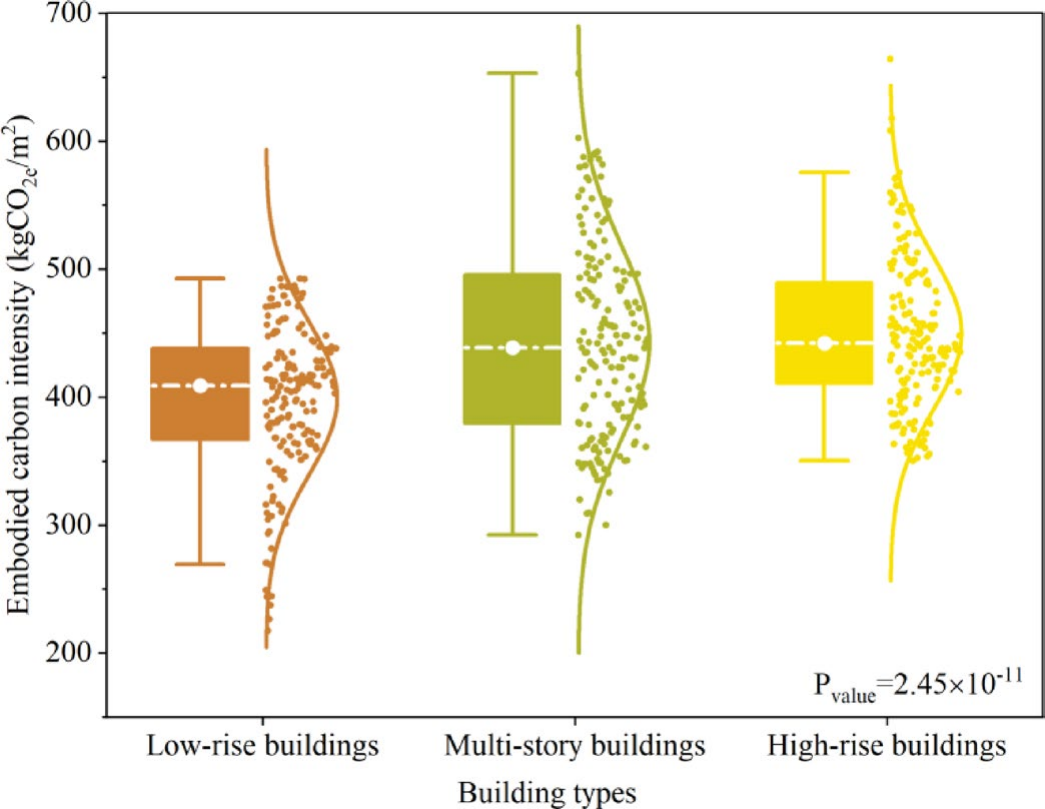
Significance test of the differences in embodied carbon intensities of low-rise, multi-story, and high-rise buildings.

Based on the above statistical analysis, the Kruskal–Wallis (K–W) test was adopted to examine whether embodied carbon intensity differed significantly among low-rise, multi-story, and high-rise buildings. As shown in Fig. 6, embodied carbon intensity differed significantly among the three building height categories at the 95% confidence level. Consequently, the 538 residential building samples were grouped by building height category for the subsequent analysis of differences in embodied carbon intensity across different characteristic categories.

### Differences in the embodied carbon intensity

**Fig. 7:**
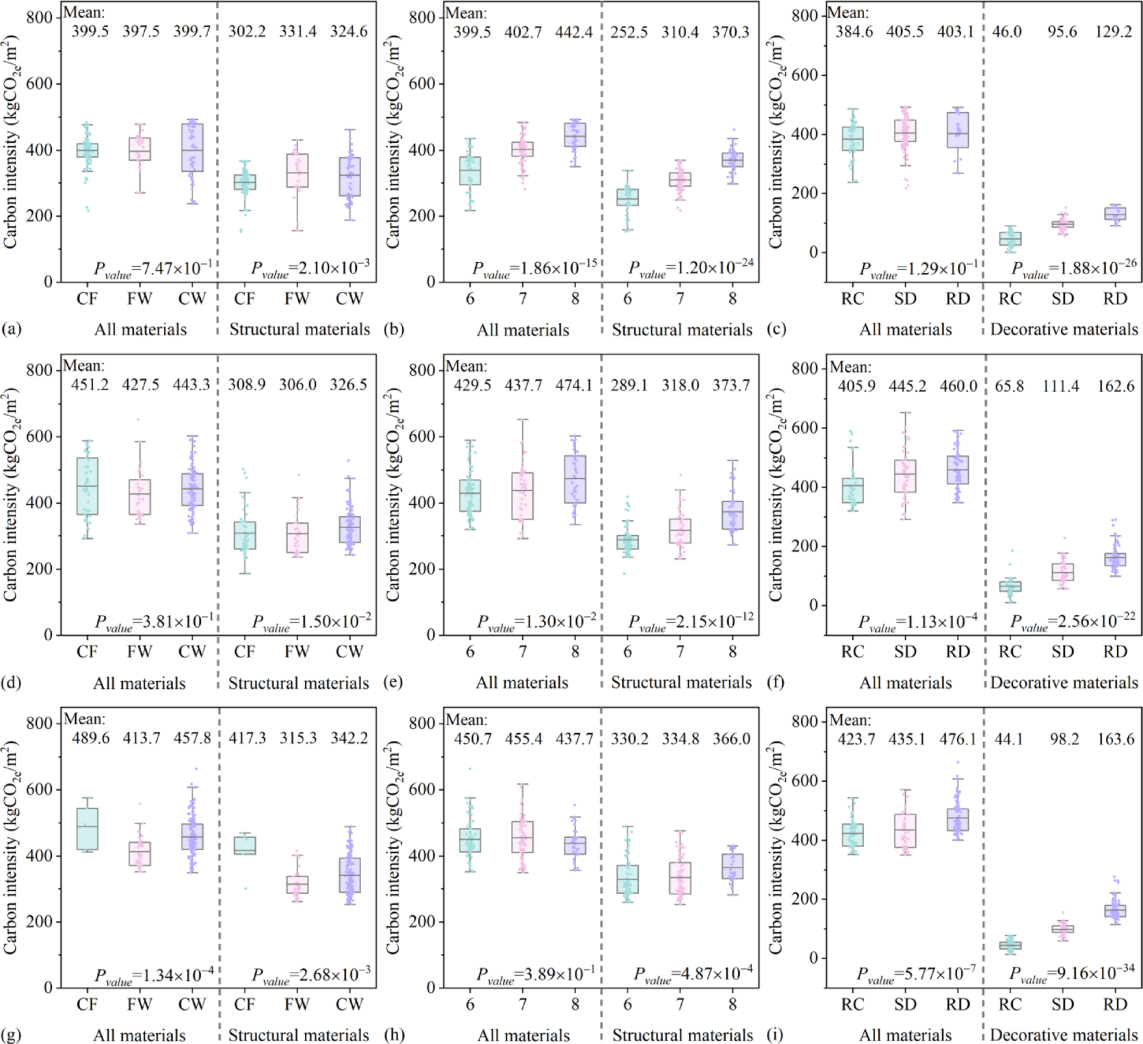
Distribution of embodied carbon intensities and significance test of the differences. (a), (b) and (c) correspond to the embodied carbon intensities clustered by the structure form, seismic fortification intensity, and delivery type in low-rise buildings; (d), (e) and (f) correspond to the relevant results in multi-story buildings; and (g), (h) and (i) correspond to the relevant results in high-rise buildings.

For low-rise, multi-story, and high-rise buildings, this study employed the K–W test to further investigate the influence of building characteristic parameters identified by correlation analysis on embodied carbon intensity. Given that structural materials were the primary contributors to embodied carbon emissions^22^, Fig. 7 presents embodied carbon intensity values calculated for 16 building material types and for structural materials alone.

Among different structural forms, high-rise buildings exhibited notable differences in total embodied carbon intensity. In contrast, only slight variations were observed in low-rise and multi-story buildings. Notably, in low-rise and multi-story buildings, while differences in total embodied carbon intensity of buildings with different structural forms were not statistically significant, the embodied carbon intensities attributed to structural materials varied significantly, with maximum differences of 29.2 and 20.5 kgCO_2e_/m^2^, respectively. This result may be partly attributed to the uneven distribution of samples with different delivery types across structural-form groups, which may weaken the differences in total embodied carbon intensity caused by structural materials. In high-rise buildings, the largest embodied carbon intensity difference was observed between frame and frame-shear wall structures, with the difference related to structural materials reaching 102.0 kgCO_2e_/m^2^.

For the building samples with varying seismic fortification intensities, total embodied carbon intensities did not show a consistent association with seismic fortification level. High-rise building samples with an 8-degree seismic fortification intensity had the lowest average embodied carbon intensity, at 437.7 kgCO_2e_/m^2^. This phenomenon may be partly attributed to the influence of variations in building decoration on the total embodied carbon intensity. Further analysis indicated that nearly 50% of the buildings with 6- and 7-degree seismic fortification intensities had refined decoration, whereas only 10% of those with an 8-degree seismic fortification intensity did. However, an overall positive correlation was noted between the embodied carbon intensities from structural materials and the seismic fortification intensity. For building clusters of different heights, differences in the embodied carbon intensity related to structural materials ranged from 35.8 to 117.8 kgCO_2e_/m^2^, with the largest difference observed in multi-story buildings.

Additionally, embodied carbon intensity varied significantly across delivery type. Embodied carbon intensity increased with higher decoration levels, and the buildings with refined decoration had significantly higher embodied carbon intensity. Moreover, the differences in the embodied carbon intensity associated with decorative materials were 83.2, 96.8, and 119.5 kgCO_2e_/m^2^, for low-rise, multi-story, and high-rise buildings, respectively. This indicated that the differences in the embodied carbon intensity related to decorative materials gradually increased with building height.

Moreover, even within the same building characteristic category, differences in embodied carbon intensity were observed among low-rise, multi-story, and high-rise buildings. As shown in Fig. 7a and d, and 7g, among the structural forms considered, t frame structures exhibited the largest differences in total embodied carbon intensity among buildings of different heights, ranging from 399.5 to 489.6 kgCO_2e_/m^2^. As shown in Fig. 7b and e, and 7h, under the same seismic fortification intensity, total embodied carbon intensity progressively increased with building height. However, the embodied carbon intensity of high-rise buildings with an 8-degree seismic fortification intensity was slightly lower than that of low-rise and multi-story buildings, partly owing to the influence of delivery type. Furthermore, at a 7-degree seismic fortification intensity, the difference in embodied carbon intensity across building height categories was the largest, reaching up to 52.7 kgCO_2e_/m^2^. As shown in Fig. 7c and f, and 7i, under the same delivery type, the embodied carbon intensities of multi-story and high-rise buildings differed only slightly but were substantially higher than those of low-rise buildings. For roughcast decoration, sample decoration, and refined decoration, the maximum differences in total embodied carbon intensity among building clusters of different heights were 18.5, 54.1, and 52.4 kgCO_2e_/m^2^, respectively. On the basis of these results, the main factors influencing the differences in embodied carbon intensity of low-rise, multi-story, and high-rise buildings are individually discussed in the next section.

### Factors influencing the differences in the embodied carbon intensity

The above results indicate that embodied carbon intensity differs significantly among certain groups classified by structural form, seismic fortification intensity, and delivery type, and that the magnitude of these differences varies across building-height categories. Accordingly, separate analyses were conducted for low-rise, multi-story, and high-rise buildings to further investigate the factors influencing differences in embodied carbon intensity under these building characteristic parameters.

#### Differences among low-rise buildings

**Fig. 8:**
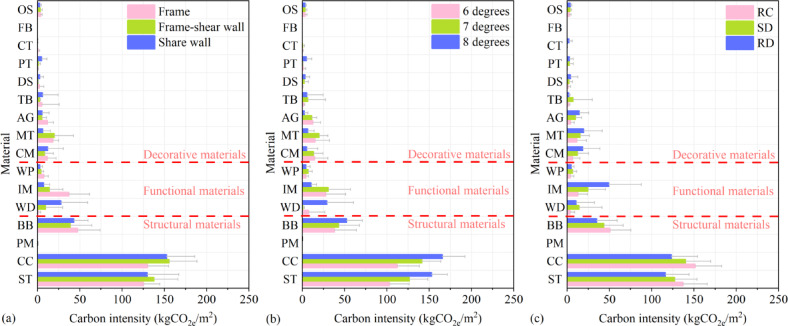
Embodied carbon intensities contributed by different types of materials in low-rise buildings: (a) comparison of different structures; (b) comparison of different seismic fortification intensities; and (c) comparison of different delivery types.

With respect to the structural form, although differences in total embodied carbon intensity were not statistically significant in the above analysis, significant differences were observed when only structural materials were considered. As shown in Fig. 8, steel and concrete were identified as the major contributors, with average influence coefficient of 0.18 and 0.48, respectively. Significant differences in total embodied carbon intensity across different seismic fortification intensities were primarily associated with concrete and steel, with influence coefficient values of 0.26 and 0.24, respectively. In terms of the delivery type, although significant differences in total embodied carbon intensity were observed, detailed analysis indicated that these variations mainly stemmed from structural materials, whereas decorative materials made a relatively smaller contribution (0.28). Among these decorative materials, cement and mortar were the main contributors.

#### Differences among multi-story buildings

**Fig. 9:**
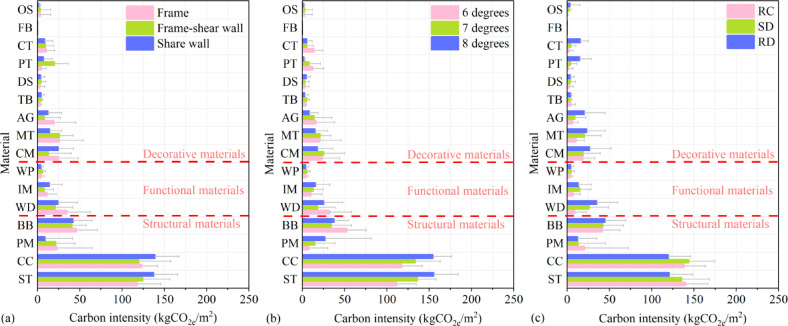
Embodied carbon intensities contributed by different types of materials in multi-story buildings: (a) comparison of different structures; (b) comparison of different seismic fortification intensities; and (c) comparison of different delivery types.

According to the significance test results, there was no significant difference in total embodied carbon intensities among multi-story buildings with different structural forms. In contrast, the embodied carbon intensities associated with structural materials showed significant variation. Specifically, steel, concrete, and prefabricated members were the primary contributors to the observed differences, with influence coefficient of 0.36, 0.31, and 0.20, respectively. As shown in Fig. 9b, structural materials were greatly associated with differences in the embodied carbon intensity among samples with varying seismic fortification intensities. Notably, steel and concrete exhibited the highest average influence coefficients (0.37 and 0.31, respectively) and were identified as the main factors influencing the observed differences. In terms of the delivery type, differences in total embodied carbon intensity were mainly associated with decorative materials rather than structural materials. On the basis of the influence coefficients, mortar, painting, and ceramic tile were the principal contributors to the observed differences, with coefficient values of 0.09, 0.08, and 0.08, respectively.

#### Differences among high-rise buildings

**Fig. 10:**
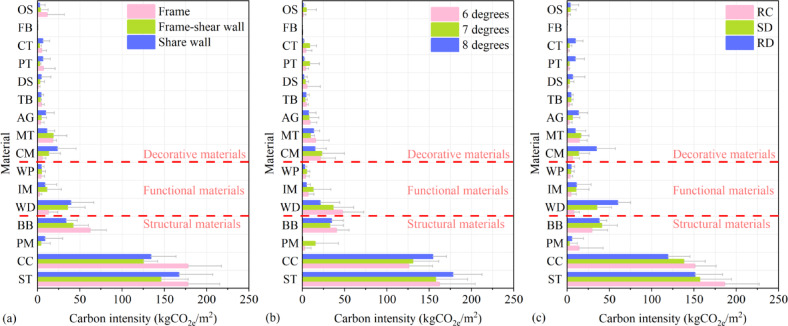
Embodied carbon intensities contributed by different types of materials in high-rise buildings: (a) comparison of different structures; (b) comparison of different seismic fortification intensities; and (c) comparison of different delivery types.

For high-rise buildings, the primary factors influencing the differences in embodied carbon intensity among structural-form groups are shown in Fig. 10a. The influence coefficients revealed that concrete, bricks and blocks, and steel together accounted for more than 50% of the total difference, with average coefficient values of 0.17, 0.23, and 0.13, respectively. Moreover, among buildings with different seismic fortification intensities, the embodied carbon intensities originating from structural materials varied substantially. Steel and concrete were identified as the major contributors, with average influencing coefficients of 0.26 and 0.36, respectively. Regarding differences in total embodied carbon intensity among buildings with different delivery types, the contribution of structural materials was slightly higher than that of decorative materials. Specifically, steel, concrete, and cement collectively accounted for 43% of the total difference.

### Discussion and suggestions

#### Differences in the contribution of major influencing factors

**Fig. 11:**
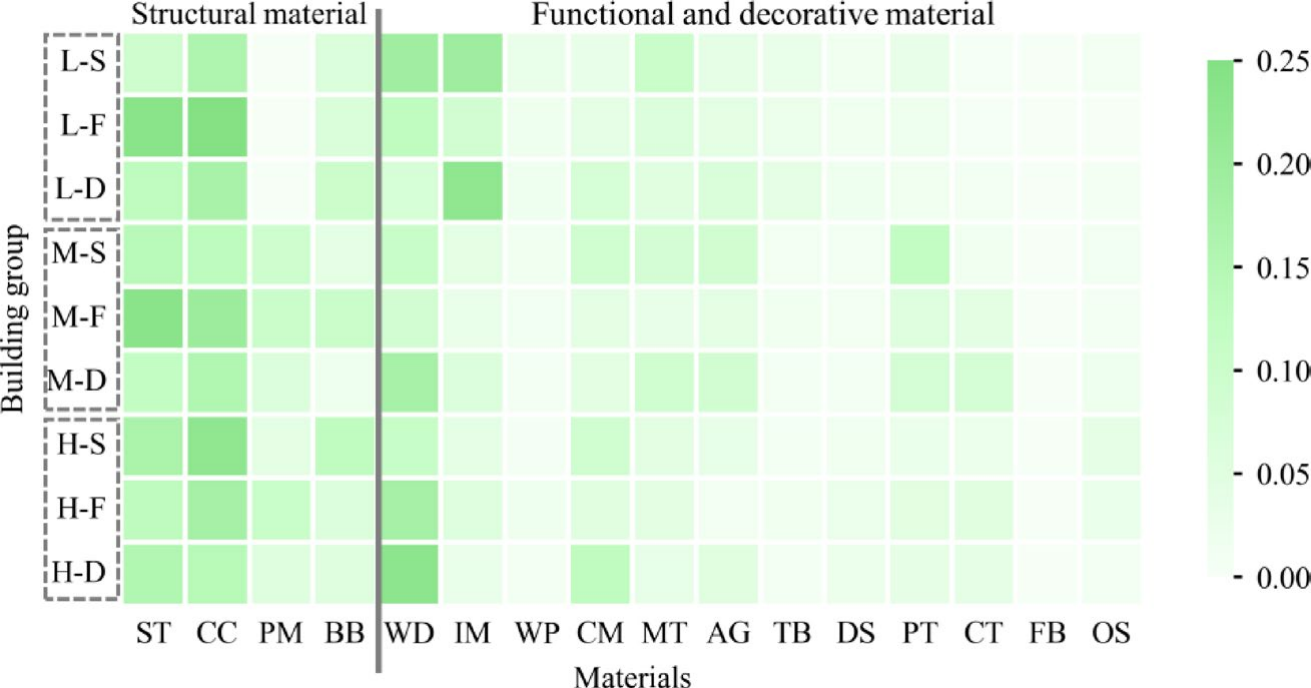
Materials contribution to the differences in embodied carbon intensities. “L-S”, “L-F”, “L-D”, “M-S”, “M-F”, “M-D”, “H-S”, “H-F, and “H-D” represent the structural forms, seismic fortification intensity and delivery type in low-rise, multi-story, and high-rise buildings, respectively.

The above results clearly indicate that the factors influencing the differences in the embodied carbon intensity across low-rise, multi-story, and high-rise buildings with various characteristic parameters were not uniform. Figure 11 shows the relative contributions of structural materials, decorative materials and functional materials to the embodied carbon intensity differences among various building clusters. The influence coefficient values of all materials for these differences are shown via color mapping.

With respect to differences in embodied carbon intensity among building samples with different structural forms, functional materials contributed more than structural materials in low-rise buildings, with average influence coefficients of 0.42 and 0.32, respectively. The underlying reason is that the buildings in cold regions typically require greater quantities of insulation materials and energy-efficient windows and doors than buildings in other regions. Among multi-story buildings with different structural forms, the observed differences in embodied carbon intensity mainly arose from structural and decorative materials. Within these categories, steel, concrete, and painting together accounted for 40% of the total contribution. Among high-rise buildings, structural materials were the major factors contributing to differences in embodied carbon intensity among different structural forms. The influence coefficients revealed that steel, concrete, and bricks and blocks were the principal contributors to these differences.

Among low-rise, multi-story, and high-rise buildings, structural materials made the largest contribution to differences in embodied carbon intensity across seismic fortification intensities, with average influence coefficients of 0.56, 0.64, and 0.48, respectively. Notably, although steel and concrete were the major contributors to differences in embodied carbon intensity across all three building types, their relative contributions differed considerably. Specifically, concrete contributed more prominently in low-rise and high-rise buildings, whereas steel was more influential in multi-story buildings.

Finally, for building samples with different delivery types, differences in embodied carbon intensity were mainly attributable to structural materials in low-rise and high-rise buildings, whereas in multi-story buildings these differences were primarily contributed by decorative materials. This indicates that although decorative materials contributed ti differences in embodied carbon intensity, their overall contribution was generally limited with that of structural materials.

#### Comparison with other studies

Table 4 presents a summary of the embodied carbon intensities of residential buildings with different heights from the literature. The results (399.2, 442.9, and 450.1, respectively) of this study agree with the ranges of values for low-rise, multi-story, and high-rise residential buildings provided in previous studies (380.0–466.3, 386.4–467.4, and 424.1–561.0, respectively), despite small differences due to different system boundaries, structural forms, and materials.

**Table 4:** The range of embodied carbon intensities in residential buildings from previous research.

Building types	Structural form	Embodied carbon (kgCO_2e_/m^2^)	System boundary^a^	Source
Low-rise building	Frame	466.3	A1-A5	Zhang et al.^37^
	Reinforced concrete	394.0 (average of 39 cases)	A1-A3	Tozan et al.^74^
	Reinforced concrete	380.0	A1-A3	Limphitakphong et al.^75^
Multi-story building	Frame	386.4	A1-A4	Gong et al.^76^
	Reinforced concrete	404.8 (average of 25 cases)	A1-A3	Luo et al.^72^
	Reinforced concrete	467.6 (average of 438 cases)	A1-A4	Zhang et al.^22^
High-rise building	Frame-shear wall	443 (average of 15 cases)	A1-A5	Cheng et al.^21^
	Reinforced concrete	561.0	A1-A5	Teng et al.^77^
	Reinforced concrete	424.1 (average of 403 cases)	A1-A4	Zhang et al.^23^

^a^ The system boundary is defined according to EN 15978:2011.

While numerous studies have focused on exploring the influences of various building characteristic parameters on embodied carbon intensities^78–81^, the underlying causes of the differences in embodied carbon intensities associated with these factors have not been thoroughly investigated. In this context, this study employed the defined influence coefficient to analyze the factors contributing to differences in the embodied carbon intensity across different building characteristic categories. Overall, steel and concrete were the primary factors driving differences in embodied carbon intensity. However, further analysis revealed that the primary factors and their contribution magnitudes differed across building height categories. For example, with respect to the differences in embodied carbon intensity among low-rise buildings with different structural forms, steel and concrete accounted for 18% and 48%, respectively, of the total difference associated with structural materials. Among multi-story buildings, steel and concrete contributed 36% and 31%, respectively, to the corresponding differences. Among high-rise buildings, steel, concrete, and bricks and blocks contributed 30%, 39%, and 23%, respectively.

#### Suggestions for embodied carbon emission mitigation

With respect to the influence of the structural form, as shown in Fig. 7a, low-rise buildings with frame structures exhibited the lowest embodied carbon intensity of 302.2 kgCO_2e_/m^2^, representing a 2–27% reduction compared with the values for multi-story and high-rise buildings. As shown in Fig. 7d, buildings with frame-shear wall structures also showed relatively low embodied carbon intensity, at 306.0 kgCO_2e_/m^2^. Furthermore, the low-carbon advantage of frame-shear wall structures was extended in high-rise buildings, with emissions decreasing by approximately 8–24%. This structural form also represents the lowest-carbon option for high-rise buildings.

With respect to seismic fortification intensity, detailed analysis showed that the low-carbon structural forms varied across different seismic fortification levels. At a 6-degree seismic fortification intensity, buildings with frame-shear wall structures exhibited the lowest embodied carbon intensity across low-rise, multi-story, and high-rise buildings compared with other structural forms. Detailed analysis indicated that the low-carbon advantage of the frame-shear wall structures was especially evident in high-rise buildings, with embodied carbon intensity reduced by 4–30% relative to the other two structural forms. For buildings designed at a 7-degree seismic fortification intensity, low-rise buildings with shear wall structures achieved the lowest embodied carbon intensity of 255.4 kgCO_2e_/m^2^. Moreover, frame structures and frame-shear wall structures can be considered viable low-carbon options for multi-story and high-rise buildings, respectively. In regions with 8-degree of seismic fortification intensity, frame structures and shear wall structures are good options for low-rise and multi-story buildings, respectively. The corresponding embodied carbon intensities were 321.9 and 365.5 kgCO_2e_/m^2^, respectively. Moreover, frame-shear wall structures showed the best low-carbon performance among the considered structural forms for high-rise buildings. It should be noted that the above analysis primarily identified low-carbon structural forms for buildings under different seismic fortification intensities primarily from a carbon-reduction perspective, without comprehensively considering the influence of other factors. Therefore, the identified low-carbon structural forms can serve as design references for reducing embodied carbon intensity. In practical engineering decision-making, structural safety, economic feasibility, and carbon-emission performance should be considered in an integrated manner.

With respect to the influence of the delivery type, although decorative materials made a limited contribution to total embodied carbon intensity, there was a notable variation in the embodied carbon intensity among buildings with different delivery types. By comparison, functional materials, including building envelope insulation and windows, and doors, deserve particular attention when aiming to reduce embodied carbon intensity. Moreover, because insulation requirements vary with building location and climate conditions, the embodied carbon intensity associated with insulation materials may differ substantially among building samples. Therefore, it is essential to select environmentally friendly building envelope materials during the design and construction phases to reduce resource consumption and embodied carbon intensity. In addition, the effects of other decorative materials, including cement, decorative panels, paint/coatings, and ceramic tiles, should be considered across all building height categories, because their cumulative contribution to embodied carbon intensity may be non-negligible. This finding is consistent with previous studies^71^. Promoting standardized decoration may therefore provide a feasible pathway for reducing decoration-related embodied carbon by minimizing material waste in typical residential buildings.

**Fig. 12:**
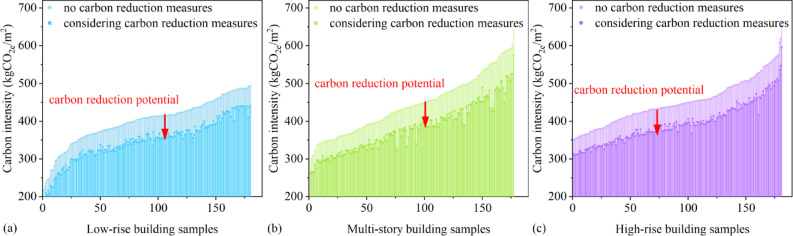
Carbon reduction potential of different building clusters.

To analyze carbon reduction benefits, two strategies, namely, optimizing material consumption and decreasing the transport distance, were considered to evaluate the carbon-saving potential of different building types. In practical applications, material consumption can be reduced by optimizing member dimensions, using high-strength reinforcement and concrete, and avoiding overdesign^38,55,82^. Transportation distances can be reduced through local procurement, optimized transportation routes, and centralized procurement and transportation^44,52^. Previous studies have verified that the consumption of structural material can be reduced by more than 10% via optimized building design^21^. When locally sourced materials are used, the transportation distance is assumed to decrease from 500 to 100 km^59^. As shown in Fig. 12, under the combined implementation of these strategies, the average carbon reduction potentials of low-rise, multi-story and high-rise buildings were 51.7, 62.0 and 56.1 kgCO_2e_/m^2^, respectively. Specifically, the maximum reduction potential was observed in multi-story buildings with frame structures, reaching 68.4 kgCO_2e_/m^2^ (15.2%). In contrast, the emissions of low-rise buildings with shear wall structures could be reduced by 47.2 kgCO_2e_ (11.8%) per square meter.

## Conclusion

In this study, statistical analysis was applied to assess the embodied carbon intensity of 538 residential buildings. Differences in embodied carbon intensity associated with various building characteristic parameters were comparatively analyzed among low-rise, multi-story, and high-rise building categories, and the main contributors to these differences were identified using the proposed influence coefficient. Targeted design recommendations were also proposed to reduce embodied carbon intensity in buildings with different height categories. The main findings are as follows:

- Analysis of the 538 residential building samples revealed that the average embodied carbon intensities of low-rise, multi-story and high-rise buildings were 399.2, 442.9, and 450.1 kgCO_2e_/m^2^, respectively. While the material production stage contributed approximately 90% of total embodied carbon intensity, the transportation stage contributed notably to its variability.
- The Kruskal‒Wallis test revealed that structural form and seismic fortification intensity significantly affected the embodied carbon intensity of structural materials, whereas delivery type was mainly associated with that of decorative materials. However, the statistical significance of differences in total embodied carbon intensity identified by single-characteristic grouping may be weakened by the combined influence of other building characteristics.
- The proposed influence coefficients indicated that although steel and concrete were the primary contributors to differences in embodied carbon intensity across buildings with different structural forms and seismic fortification intensities, their relative contributions varied across building height types. Concrete was the dominant factor in low-rise and high-rise buildings, while steel made a greater contribution in multi-story buildings.
- Design recommendations for low-carbon residential buildings were proposed from the perspective of embodied carbon reduction. frame-shear wall structures showed relatively favorable low-carbon performance across all building height categories only in regions with a 6-degree seismic fortification intensity. However, in regions with seismic fortification intensities of 7 and 8 degrees, the low-carbon structural forms varied among building height categories.
- Based on comparisons of among different building clusters, low-rise and multi-story buildings with frame structures exhibited significant carbon reduction potential, possibly achieving reductions of 13.9% and 15.2%, respectively, through a combined strategy involving material usage optimization and transportation distance reduction. Among high-rise buildings, shear wall structures provided the highest carbon reduction potential, at 12.5%.

In summary, differences in embodied carbon intensity among low-rise, multi-story, and high-rise buildings under the influence of various building characteristic parameters were analyzed in depth. The findings can support the evaluation of carbon emission benchmarks for residential buildings and provide practical guidance for low-carbon building design. However, several limitations should be address in future research. First, including more samples of buildings with refined decoration could improve the uniformity of the sample distribution and thereby enhance the reliability of statistical results. Second, analyzing differences in embodied carbon intensity based on a single building characteristic cannot fully isolate the combined influence of other building characteristics. Further research could adopt decoupling analysis approaches to further explore the statistical characteristics of embodied carbon intensity in residential buildings. Finally, by expanding the building case database, the common characteristics of low-carbon buildings can be further explored in terms of design strategies, material selection, construction techniques, and cost-effectiveness, thereby providing more comprehensive and economically feasible emission reduction pathways for residential buildings.

## Data Availability

The datasets generated during and/or analyzed during the current study are available from the corresponding author on reasonable request.

## Abbreviations

CF: Frame structure
FW: Frame-shear wall structure
CW: Shear wall structure
RC: Roughcast
SD: Simple decoration
RD: Refined decoration
IF: Isolated foundation
RF: Raft foundation
MF: Mat foundation
TF: Strip foundation
PF: Pile foundation
OT: Other foundation
HC: Central China
HN: North China
HE: East China
HS: Southern China
NW: Northwestern china
SW: Southwestern China
NE: Northeastern China
